# PCR-based detection of *Plasmodium *in *Anopheles *mosquitoes: a comparison of a new high-throughput assay with existing methods

**DOI:** 10.1186/1475-2875-7-177

**Published:** 2008-09-15

**Authors:** Chris Bass, Dimitra Nikou, Andrew M Blagborough, John Vontas, Robert E Sinden, Martin S Williamson, Linda M Field

**Affiliations:** 1 Center for Sustainable Pest and Disease Management, Department of Biological Chemistry, Rothamsted Research, Harpenden, AL5 2JQ, UK; 2 Vector Group, Liverpool School of Tropical Medicine, Pembroke Place, Liverpool, L35QA, UK; 3 Department of Biological Sciences, Imperial College, London, UK; 4 Laboratory of Pesticide Science, Agricultural University of Athens, Iera Odos 75, 118 55, Votanikos, Athens, Greece

## Abstract

**Background:**

Detection of the four malaria-causing *Plasmodium *species (*Plasmodium falciparum*, *Plasmodium vivax*, *Plasmodium ovale *and *Plasmodium malariae*) within their mosquito hosts is an essential component of vector control programmes. Several PCR protocols have been developed for this purpose. Many of these methods, while sensitive, require multiple PCR reactions to detect and discriminate all four *Plasmodium *species. In this study a new high-throughput assay was developed and compared with three previously described PCR techniques.

**Methods:**

A new assay based on TaqMan SNP genotyping was developed to detect all four *Plasmodium *species and discriminate *P. falciparum *from *P. vivax*, *P. ovale *and *P. malariae*. The sensitivity and the specificity of the new assay was compared to three alternative PCR approaches and to microscopic dissection of salivary glands in a blind trial of 96 single insect samples that included artificially infected *Anopheles stephensi *mosquitoes. The performance of the assays was then compared using more than 450 field-collected specimens that had been stored on silica gel, in ethanol or in isopropanol.

**Results:**

The TaqMan assay was found to be highly specific when using *Plasmodium *genomic DNA as template. Tests of analytical sensitivity and the results of the blind trial showed the TaqMan assay to be the most sensitive of the four methods followed by the 'gold standard' nested PCR approach and the results generated using these two methods were in good concordance. The sensitivity of the other two methods and their agreement with the nested PCR and TaqMan approaches varied considerably. In trials using field collected specimens two of the methods (including the nested protocol) showed a high degree of non-specific amplification when using DNA derived from mosquitoes stored in ethanol or isopropanol. The TaqMan method appeared unaffected when using the same samples.

**Conclusion:**

This study describes a new high-throughput TaqMan assay that very effectively detects the four *Plasmodium *species that cause malaria in humans and discriminates the most deadly species, *P. falciparum*, from the others. This method is at least as sensitive and specific as the gold standard nested PCR approach and because it has no requirement for post-PCR processing is cheaper, simpler and more rapid to run. In addition this method is not inhibited by the storage of mosquito specimens by drying or in ethanol or isopropanol.

## Background

A principal component in malaria vector control and monitoring programmes is the sensitive detection of human-specific *Plasmodium *species in the mosquito host. The infection status of a mosquito is usually assessed by the presence/absence of *Plasmodium *sporozoites in the salivary glands. Traditionally, this was done by dissection and visual assessment of glands using a microscope. However, this requires skilled personnel, is time consuming and does not determine which *Plasmodium *species is present. It has, therefore, been largely superseded by more rapid immunological and molecular approaches. One of the most widely adopted of these higher-throughput approaches is the circumsporozoite protein enzyme-linked immunosorbent assay (CSP ELISA) [1,2]. However, although CSP ELISA has proven to be relatively robust and cheap, there are a number of potential drawbacks in using this approach. Firstly, there have been several reports that CSP ELISA overestimates true salivary gland infection rates and this may be linked to the spread of circumsporozoite protein throughout the mosquito after being shed from sporozoites while migrating through the mosquito [3-6]. Secondly, mosquitoes often need to be collected and stored for later analysis and this is usually done by either drying on silica gel or keeping in ethanol or isopropanol. While the former is not inhibitory to ELISA the latter approach renders the specimens unsuitable for ELISA testing. Thirdly, although monoclonal antibodies have been produced for all four *Plasmodium *species that cause human malaria each assay must be run separately and therefore in practice many studies only test for the presence of one or two of the species. Finally the CSP ELISA may also be relatively insensitive to very low-level infections [7].

PCR-based assays are usually an order of magnitude more sensitive than ELISA and several have been described for the detection of *Plasmodium *in mosquitoes. Many utilize primers designed against species specific regions in the sequences encoding the small subunit ribosomal RNA (ssrRNA) to detect *Plasmodium falciparum *or all four *Plasmodium *species [8-10]. To enhance sensitivity further a nested approach using two rounds of PCR may be employed as is the case with the assay designed by Snounou *et al *which is now widely regarded as a 'gold standard' for PCR-based detection of *Plasmodium *species [11]. However, a significant disadvantage of these PCR approaches is the requirement for separate PCR reactions for each species and for post-PCR processing (gel electrophoresis of PCR products).

More recently, high-throughput assays based on real-time PCR have been developed for detection of the malaria parasite. These include real-time PCR using either SYBR green dye or TaqMan probes and real-time Nucleic Acid Sequence-Based Amplification (NASBA) [12-14]. Currently, these methods have not been tested for detection of sporozoites in the mosquito host and require multiple reactions to detect all four species.

The aim of this study was to develop a PCR-based method that 1) can detect all four *Plasmodium *species in the mosquito vector and discriminate between *P. falciparum *and the other three species in a single reaction, 2) is as sensitive as the gold standard nested PCR approach, 3) will not be inhibited by the storage of mosquito specimens by drying or in ethanol/isopropanol 4) will be as cheap or cheaper to run than standard PCR. These aims were addressed by developing a high-throughput 'closed-tube' approach based on TaqMan SNP genotyping.

## Methods

### Plasmodium genomic DNA samples and plasmid clones

*Plasmodium *genomic DNAs of diverse geographic origin (MRA numbers, 102G, 149G, 150G, 152G, 153G, 157G, 159G, 160G, 161G, 163G, 165G, 167G, 168G, 176G, 200G, 201G, 202G, 273G, 274G, 275G, 276G, 340G, 341G) and four plasmid clones (MRA number 177–180) carrying a PCR insert of the ssrRNA gene amplified from either *P. falciparum*, *Plasmodium vivax*, *Plasmodium malariae *or *Plasmodium ovale *were obtained from the Malaria Research and Reference Reagent Resource Center (ATCC, Manassas, Virgnia, USA). Additional *Plasmodium *DNAs were kindly provided by Dr Debbie Nolder at the HPA Malaria Reference Laboratory, London School of Hygiene & Tropical Medicine, UK. To determine the sensitivity of each of the PCR methods a dilution series of three DNA samples for each of the four human *Plasmodium *species was used. For this, the DNAs were diluted to 20 ng/μl (as determined by absorption at 260 nm using a NanoDrop spectrophotometer, NanoDrop Technologies, Wilmington, DE) and then serial dilutions were made down to 1 in 1 × 10^6 ^(20 fg/μl).

### Mosquito specimens and blind trial

The PCR methods described in this study were tested in a blind trial using artificially infected *Anopheles stephensi*. For this mature *P. falciparum *3D7 gametocytes were cultured in continuous culture apparatus as described previously [15] for periods of 14–18 days at 37°C under 3% O_2_, 5% CO_2_, 92% N_2 _continuous gas flow. The initial haematocrit of cultures was 7% (v/v) RBC in RPMI medium (Gibco), containing 2 g/l NaHCO_3_, 25 mM HEPES, 50 μg/ml hypoxanthine and 10% (v/v) human group 'AB' heat inactivated serum and the medium was changed every 12 hours. After 14 days, gametocyte quality and quantity was tested every 24 hours by giemsa smears and an exflagellation test. Fifty fields of a monolayer of infected erythrocytes were examined at 40 × magnification by phase-contrast optics and the numbers of centres of exflagellation following incubation at 24°C for 20 mins were recorded. Mature cultures showing high levels of exflagellation were identified, harvested and fed to *An. stephensi *mosquitoes. During this process, gametocyte cultures were spun at 500 g for 4 min, the supernatant removed and the pellet containing parasitized red blood cells was mixed with 700 μl of freshly washed and pre-warmed uninfected RBC. 1 ml of pre-warmed heat inactivated AB serum was then added to this mixture, which was then fed to pots of 50 mosquitoes (starved 5–6 hours pre-feed) via 500 μl mini-feeders, as described previously [16]. Mosquitoes were allowed to engorge on each blood feed for 20 min through a Parafilm membrane, warmed to 37°C using a water jacket. After feeding, mosquitoes were kept at 25–26°C, 80 % humidity with access to 5% glucose, 0.05 % para-aminobenzoic acid (PABA) solution. Individual mosquitoes were assessed for the presence of sporozoites by salivary gland dissection in TE buffer at 16 days post-feed. The dissected glands and remains of the head and thorax in 100 μl TE buffer were then frozen at -20°C until use. The trial plate consisted of eighty four mosquitoes that had been dissected and scored as either sporozoite positive (n = 36) or negative (n = 48). As low level infections can be easily missed by dissection [17] an additional ten mosquitoes that had no prior exposure to *P. falciparum *were also dissected in the same way and included in the trial as negative controls. Two buffer only wells were also included as additional negative controls. The samples were heated to 99°C for ten minutes to release the DNA from sporozoites then cooled on ice and five μl used as template in PCR.

PCR methods were subsequently tested with 483 field-collected mosquitoes collected from Equatorial Guinea, Ghana, Kenya, and Mozambique. These were morphologically identified as *Anopheles gambiae sensu lato *(s.l) or *Anopheles funestus *before storing on silica gel, in 70% ethanol, or in isopropanol. Mosquitoes from each storage condition were divided into two groups and then DNA extracted from the head and thorax of single mosquitoes using either the Livak or DNAzol methods [18,19]. Mosquitoes were identified to species by allele-specific PCR [20,21] or a new TaqMan protocol [22]. The trial of PCR methods using these samples comprised of 263 *An. gambiae *individuals, 184 *An. funestus *individuals and 36 *An. arabiensis *individuals and five water negative controls.

### Statistical methods

Agreement between the results of the TaqMan assay and the nested PCR 'gold standard' reference method was measured using Cohen's Kappa measure of test association [23].

### Snounou single and nested PCRs

Single and nested PCRs were carried out as described previously [9,11].

### TaqMan assay

Nucleotide alignment of the small subunit ribosomal RNA gene sequences of the four human infecting *Plasmodium *species available in the National Center for Biotechnology Information (NCBI) database revealed a region that contained two single nucleotide polymorphisms (SNPs) four base pairs apart specific to *P. falciparum *flanked by an area of conserved sequence. Two minor groove binding (MGB) probes (Applied Biosystems) that bind over both SNPs were designed using the Primer Express™ Software Version 2.0. The probe Falcip+ (5'-TCTGAATACGAATGTC-3') was labelled with 6-FAM at the 5' end for the detection of *P. falciparum *and the probe OVM+ (5'-CTGAATACAAATGCC-3') was labelled with VIC for the detection of either *P. malariae*, *P. ovale *or *P. vivax*. Each probe also carried a 3' nonfluorescent quencher and a minor groove binder at the 3' end. The minor groove binder provides more accurate allelic discrimination by increasing the T_M _between matched and mis-matched probes [24]. Forward, PlasF (5'-GCTTAGTTACGATTAATAGGAGTAGCTTG-3') and reverse, PlasR (5'-GAAAATCTAAGAATTTCACCTCTGACA-3') primers that flank the probe binding site were also designed using the Primer Express software.

PCR reactions (20 μl) contained 1 μl of genomic DNA, 10 μl of SensiMix DNA kit (Quantace), 800 nM of each primer and 300 nM of probe PlasF and 200 nM of probe OVM+. Reactions were run on a Rotor-Gene 6000™ (Corbett Research) using the temperature cycling conditions of: 10 minutes at 95°C followed by 40 cycles of 95°C for 10 seconds and 60°C for 45 seconds. The increase in VIC and FAM fluorescence was monitored in real time by acquiring each cycle on the yellow (530 nm excitation and 555 nm emission) and green channel (470 nm excitation and 510 emission) of the Rotor-Gene respectively.

### Tassanakajon PCR

PCR was carried out as described previously [10] except for modification of the annealing temperature from 45°C to 50°C.

## Results

### Snounou nested and single PCRs

**Figure 1:**
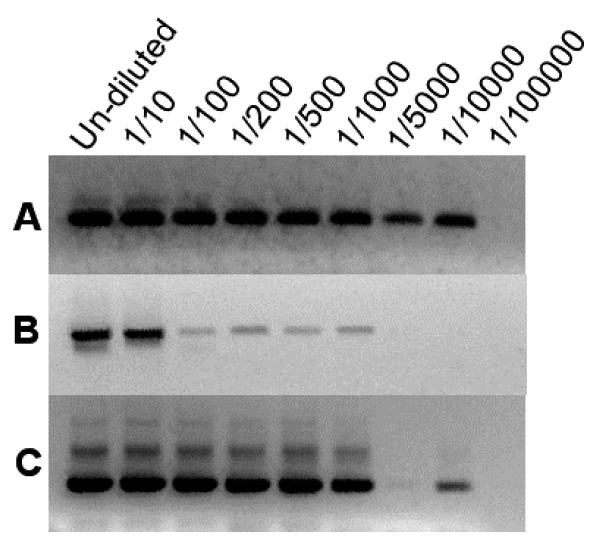
**Sensitivity of PCR detection assays for *P. falciparum***. The dilution of the original 20 ng DNA template is given above each lane. (A) Snounou nested PCR assay, (B) Snounou single PCR assay, (C) Tassanakajon PCR assay.

The nested and single PCRs described by Snounou et al [9,11] were optimized using *Plasmodium *genomic DNA as template and the sensitivity was compared to the other assays using the same set of DNA dilutions of each of the four *Plasmodium *species (an example using dilutions of *P. falciparum *DNA is shown in Figure 1). The nested PCR protocol showed a limit of detection for *P. falciparum *and *P. vivax*, at a 1 :10,000 dilution representing a low limit detection of 2 picograms of DNA. The limit of detection for *P. ovale *was a 1:100,000 dilution (0.2 pg DNA) and for *P. malaria *a 1:100 (0.2 ng DNA) dilution. The single PCRs showed a low limit detection of 1:5,000 (4 pg DNA) for *P. falciparum *and *P. vivax *and 1: 1,000 (20 pg DNA) for *P. ovale *and 1:10 (2 ng DNA) for *P. malariae*. The two PCR methods were then tested in a blind trial of 96 samples that included mosquitoes artificially infected with *P. falciparum*. The results are shown in full in Additional file 1 and summarized in Table 1. Both methods scored the ten '*P. falciparum *un-exposed negative' mosquito samples and the buffer blanks as negative. Of the 36 samples which had scored positive by dissection, 33 also scored positive by nested PCR and of the 48 samples which had scored negative by dissection 10 were scored positive by nested PCR. The single PCR approach failed to detect *P. falciparum *in any of the samples.

**Table 1:** Summary of the results of the blind trial using artificially infected *Anopheles stephensi*

	**Dissection**	**TaqMan**	**Snounou nested**	**Tassanakajon**	**Snounou single**
**Number of negative controls scored positive**	0	0	0	0	0
**Number of samples positive by dissection scored positive**	36	35	33	30	0
**Number of samples negative by dissection scored positive**	0	12	10	8	0

**Figure 2:**
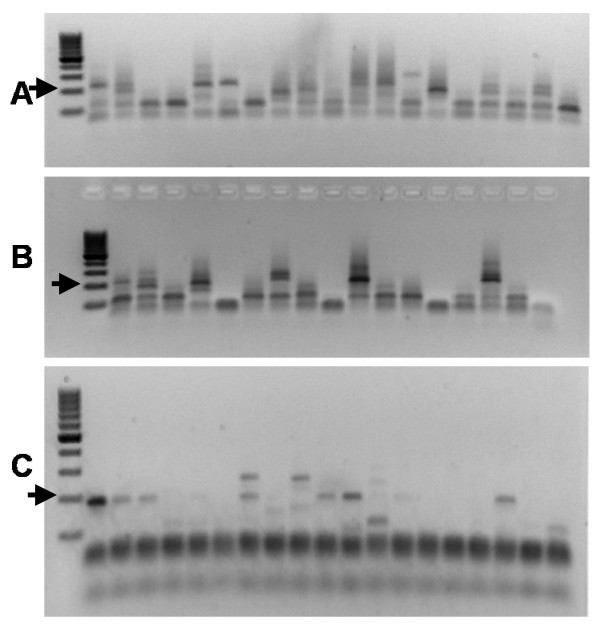
**Non-specific bands produced by PCR detection assays**. (A) The Snounou nested approach using DNA derived from mosquitoes stored in isopropanol, (B) the Snounou nested approach using DNA derived from mosquitoes stored in ethanol, (C) the Tassanakajon PCR using DNA derived from mosquitoes stored on silica gel. In each gel the expected diagnostic band size is indicated by an arrow.

Both PCR methods were then tested using DNA extracted from wild-caught *An. gambiae s.s*., *An. arabiensis *and *An. funestus *specimens that had been stored on silica gel, in ethanol or in isopropanol (Additional file 2 and Table 2). The single PCR approach was unable to detect *Plasmodium *in the first 192 samples tested and therefore the decision was made to abandon this method due to its apparent lack of sensitivity. The nested PCR approach recorded 12 *P. falciparum *positive and 1 *P. vivax *positive specimens from the DNAs of the 269 silica-dried mosquitoes. However PCR using the 215 DNA samples derived from mosquitoes stored in ethanol or isopropanol produced a high number of non-specific PCR products when screening for *P. falciparum *(Figure 2) preventing unambiguous scoring of these samples which were therefore designated as 'failed reactions'.

**Table 2:** The level of agreement between the different PCR assays and the 'gold standard' Snounou nested PCR in trials with artificially infected and field-collected mosquitoes as determined by Cohen's Kappa measure of test association

	Artificially infected	Field-collected
	
Method	n = 96	n = 269^a^
Snounou nested	κ = 1	κ = 1
TaqMan	κ = 0.858	κ = 0.925
Tassanakajon	κ = 0.767	κ = 0.264

^a^These samples do not include those stored in ethanol/isopropanol

### TaqMan assay

**Figure 3:**
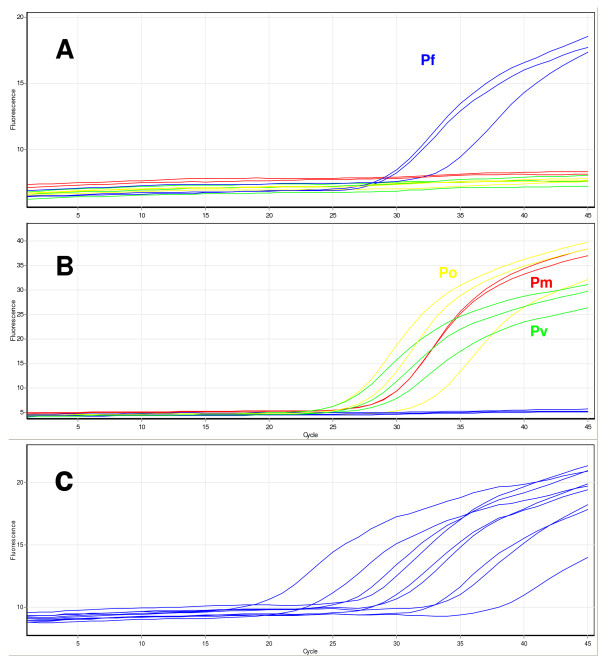
**Detection of *Plasmodium *species using the newly developed TaqMan assay**. In this example two or more specimens of *P. falciparum *(blue trace), *P. vivax *(green trace), *P. ovale *(yellow trace) and *P. malariae *(red trace) were tested. Part A displays the cycling of the FAM-labelled probe specific to *P. falciparum*. Part B displays the cycling of the VIC-labelled probe specific to *P. malariae*, *P. ovale *and *P. vivax*. Part C displays a dilution series of *P. falciparum *DNA, from left to right traces are generated from diluted DNA of the following concentrations: 1:10, 1:100, 1:200, 1:500, 1:1000, 1:2000, 1:5,000, 1:10,000 and 1:100,000.

**Figure 4:**
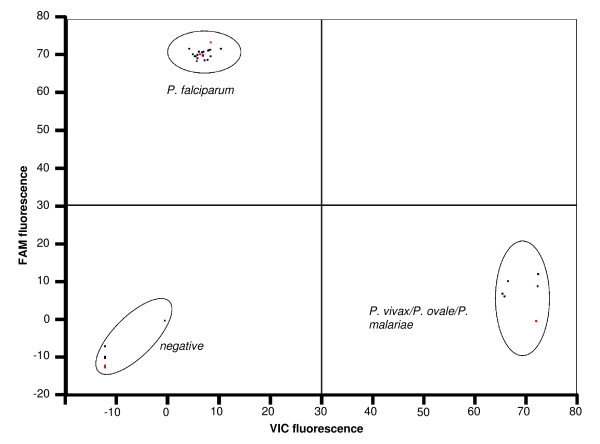
**Scatter plot analysis of TaqMan fluorescence data**. In this example the TaqMan assay was carried out on ~30 Plasmodium genomic DNA samples and five no template negative controls. Fluorescence values of the FAM labelled probe specific for *P. falciparum *were plotted against those of the VIC labelled probe specific for *P. vivax, P*. ovale and *P. malariae*.

After minimal optimisation the TaqMan assay was able to detect all four human *Plasmodium *species using genomic DNA as template. This assay uses two probes, the first labelled with 6FAM detects *P. falciparum *and the second, labelled with VIC, detects *P. vivax*/*P. ovale*/*P. malariae*. Thus, a substantial increase in FAM fluorescence during PCR indicates the presence of *P. falciparum *whilst a substantial increase in VIC fluorescence indicates the presence of *P. vivax*, *P. ovale *or *P. malariae *as shown in Figure 3. An increase in both dyes would indicate a mixed infection. To help with species assignment the Rotor-Gene software allows endpoint fluorescence values for the two dyes to be automatically corrected for background and plotted against each other in bi-directional scatter plots (for an example see Figure 4). Tests of the assay using a range of *Plasmodium *genomic DNA samples and plasmid clones carrying a PCR insert of the ssrRNA gene showed the new method to be specific with all samples correctly scored (n = 36). This also confirmed that the two SNP sites over which the probes were designed are conserved in *P. falciparum *from several geographic regions. The analytical sensitivity of the TaqMan method was examined using a dilution series of three DNA samples for each of the four *Plasmodium *species. The limit of detection for each species was a 1:100,000 dilution equivalent to 0.2 picograms of DNA which is approximately 10 parasite genomes (an example using dilutions of *P. falciparum *DNA is shown in Figure 3). The performance of the optimized TaqMan assay was assessed in a blind trial of 96 samples that included *An. stephensi *mosquitoes artificially infected with *P. falciparum*. The results are shown in full in Additional file 1 and summarized in Table 1. The TaqMan assay scored the ten 'non-exposed negative' mosquito samples and the buffer controls as negative. Of the 36 samples assessed as positive by dissection 35 scored positive by TaqMan and of the 48 samples scored negative by dissection 12 scored positive by TaqMan. Thus close agreement was seen between the results of TaqMan and the 'gold standard' nested PCR approach (κ = 0.858).

Finally the TaqMan assay was tested using DNA extracted from wild-caught *An. gambiae s.s*., *An. arabiensis *and *An. funestus *specimens that had been stored on silica gel or in ethanol or isopropanol. Of the 483 samples 31 (6%) tested positive for *P. falciparum *and one sample (0.2%) tested positive for *P. vivax*/*P. ovale*/*P. malariae *(see Additional file 2 and Table 2). Of the positive samples 18 were *An. gambiae s.s*., 7 were *An. funestus *and 7 were *An. arabiensis*. The assay did not appear to be affected by any of the storage methods. When the results using the TaqMan method were compared with the Snounou nested PCR reference they were shown to be highly concordant, κ = 0.925 (this comparison did not include the samples stored in isopropanol/ethanol due to the failure of these samples in nested PCR).

### Tassanakajon PCR

The PCR protocol of Tassanakajon *et al *[10] was optimized using *Plasmodium *genomic DNA as template. This method is designed to detect only *P. falciparum *and the sensitivity using dilutions of *P. falciparum *DNA showed the limit of detection to be a 1 in 10,000 dilution (Figure 1) representing a low limit detection of 2 picograms of DNA. The performance of the method was assessed in a blind trial of 96 samples that included mosquitoes that had been artificially infected with *P. falciparum*. The results are shown in full in Additional file 1 and summarized in Table 1. The Tassanakajon assay scored the ten 'non-exposed negative' mosquito samples and the buffer controls as negative. Of the 36 samples scored positive by dissection 30 scored positive by Tassanakajon PCR and of the 48 samples scored negative by dissection 8 scored positive by Tassanakajon PCR.

The results of this method to test DNA extracted from wild-caught *An. gambiae s.s*., *An. arabiensis *and *An. funestus *specimens that had been stored on silica gel or in ethanol/isopropanol are shown in Additional file 2 and summarized in Table 2. A total of 53 of the 483 samples (11%) tested positive for *P. falciparum*. However, some non-specific PCR products were present in some of the samples analysed (including those stored on silica gel) using this method (Figure 2) which made the results more difficult to interpret. Attempts were made to improve the specificity of the PCR by modifying the annealing temperature in the original protocol from 45°C to 50°C, however, this did not completely eliminate the problem. The difficulties in interpreting some of the PCRs may partially explain the low agreement between this method and the nested PCR reference (κ = 0.264). In addition, no *Plasmodium *was detected in any of the 93 DNA samples from mosquitoes stored in ethanol.

## Discussion

Determination of sporozoite rates in mosquito vectors is an important component of malaria control programmes, allowing the assessment of transmission risk and the effect of any control interventions. Because the sporozoite burden of individual mosquitoes can vary [3], without necessarily affecting infectivity, and because storage of mosquitoes may reduce the quantity and/or quality of the extracted DNA, sensitive detection methods are required. In this study the ability of a new assay and three existing PCR-based approaches to detect sporozoites in the mosquito vector were compared. The nested PCR approach described by Snounou *et al *[11] which is regarded as the 'gold-standard' proved to be sensitive and specific in these trials. When the performance of this assay using artificially infected mosquitoes was compared with microscopic dissection three of the thirty six samples assessed as *P. falciparum *positive by dissection were scored negative by nested PCR. This low number of false negatives could have been due to sample degradation following dissection. Significantly, ten of the samples assessed as *P. falciparum *negative by dissection were scored positive by this method. It is likely these additional positive samples are genuine as sporozoite detection by dissection has a low sensitivity and low level infections are often missed [17]. For this reason the trial also included ten mosquito samples that had never been exposed to *P. falciparum *as true negatives and these were all scored negative by the nested PCR assay. The nested method also performed well on DNAs derived from mosquito specimens that had been stored on silica gel. However, when using DNA derived from mosquito specimens that had been stored in ethanol or isopropanol no usable results were obtained due to the presence of non-specific PCR products. It is possible that alternative DNA extraction methods might overcome this as a study examining five alternative extraction methods showed variation in sensitivity/inhibition using the different approaches [7].

In comparison with the nested approach the Snounou single PCRs performed less well. Tests of this method using *Plasmodium *genomic DNA dilutions showed the assay to be at least a factor of ten less sensitive than the nested approach. The reduced sensitivity of this method was reflected in the trials with artificially infected and wild-caught mosquitoes where no positive samples were recorded. These results indicate that this method has insufficient sensitivity to routinely detect sporozoites in mosquitoes.

Two disadvantages of the nested PCR approach are the requirement for two rounds of PCR, which lowers throughput and increases assay run cost, and the requirement for separate PCRs for each species. To test for *P. falciparum *using this approach costs approximately US$ 0.88 per specimen analysed, to test for all four species costs approximately US$ 2.2. Because of the drawback of the nested PCR approach an alternative PCR method that requires only a single round of PCR was also investigated. Tests of the method of Tassanakajon *et al *[10] using *P. falciparum *genomic DNA dilutions showed the assay to have sensitivity comparable to the Snounou nested PCR. Indeed when the performance of this assay was compared to the nested PCR approach using artificially infected mosquitoes the two were in good agreement (κ = 0.766) although a higher number of false negatives (samples scored positive by dissection scored negative by PCR) was observed (six compared to three by nested PCR) suggesting a slightly lower overall sensitivity. The Tassanakajon PCR method performed less well on DNAs derived from mosquitoes that had been stored on silica gel or in isopropanol or ethanol where interpreting the results was hindered by the presence of non-specific bands in some samples. Overall this method scored many more samples *P. falciparum *positive than the Snounou nested assay when using DNA from mosquitoes stored on silica gel. It is likely these additional positive samples are false positives as the trial using artificially infected mosquitoes indicated a lower sensitivity of detection than the nested approach. This method scored no samples positive when using DNA derived from mosquitoes stored in ethanol indicating that this storage method may not be suitable for this assay. An additional disadvantage of the Tassanakajon method is that it is specific for *P. falciparum *and the other three species infecting humans can not be detected using this approach.

To overcome the disadvantages of the existing PCR protocols a 'closed tube' approach that requires no post-PCR processing of samples was developed. This assay was designed to detect all four *Plasmodium *species whilst also discriminating *P. falciparum *from the other species in a single reaction. The rationale behind this approach is that it allows *P. falciparum *which is responsible for most of the severe morbidity and mortality caused by malaria world-wide and is by far the most prevalent species in sub-Saharan Africa [25] to be distinguished from the other species. The TaqMan method proved to be highly specific using genomic DNA samples for all four *Plasmodium *species and was also the most sensitive of all the assays trialled. This was reflected in the blind trial using artificially infected mosquitoes where the TaqMan assay showed the lowest number of false negatives, showed very close agreement with the nested PCR approach and, unlike the latter, displayed no reduction in sensitivity when testing DNA derived from mosquitoes stored in ethanol or isopropanol. The cost of this assay as performed in this study was approximately US$ 0.73 per sample. Additional experiments were carried out with the same DNA dilutions to see if reagent volumes could be reduced without affecting the sensitivity of the assay. Using the real-time PCR machine described in this study no loss of sensitivity was observed for half volumes of reagents, this further reduces assay cost to approximately US$ 0.5 per sample. The one disadvantage of the TaqMan assay is the initial cost of the real-time PCR machine, which is in the region of US$ 19,000 to 25,000, compared to the start-up cost of conventional PCR of around US$ 10,000 [18]. However, the price of real-time PCR machines are falling and in the future is likely to become closer to standard thermocyclers. In this study the performance of the TaqMan assay was not assessed using pools of several mosquitoes. If future studies prove the reliability of this method on pools of mosquitoes further increases in throughput and reductions in costs may be possible.

## Conclusion

In summary, in tests of PCR methods for the detection of *P. falciparum*, *P. vivax*, *P. ovale *and *P. malariae *the new TaqMan method showed sensitivity and specificity at least as good as the gold standard nested PCR approach and unlike the latter was not inhibited by the storage of mosquito specimens by drying or in ethanol/isopropanol. The new method is cheaper to run than nested PCR and simple and rapid to perform due to the high-throughput and closed-tube nature of the TaqMan approach.

## Competing interests

The authors declare that they have no competing interests.

## Authors' contributions

CB developed the TaqMan assay, optimized and ran the previously described PCR methods and drafted the manuscript. DN helped to design and optimize the assays and helped draft the manuscript. AMB prepared the artificially infected mosquitoes and helped draft the manuscript. JV, RES, MSW and LF, helped draft and critically review the manuscript. All authors read and approved the final manuscript.

## Supplementary Material

Additional file 1 **Excel spreadsheet showing the full results of the trial of PCR methods for detection of *P. falciparum *using artificially infected *An. stephensi *mosquitoes**. Mosquito samples that had no exposure to *P. falciparum *and buffer negative controls are highlighted in grey. A *P. falciparum *positive result is indicated by (Pf+) and a negative result by (-). Click here for file

Additional file 2 Excel spreadsheet showing the full results of the trial of PCR methods for detection of *P. falciparum *(Pf), *P. vivax *(Pv), *P. malariae *(Pm) and *P. ovale *(Po) using wild caught *An. gambiae s.s*., *An. arabiensis *and *An. funestus *mosquitoes. Click here for file
